# PP32 Ultra-Fast Health Technology Assessments: A Descriptive Analysis Of Practices In A Latin American Agency

**DOI:** 10.1017/S0266462325102511

**Published:** 2025-12-29

**Authors:** Carla Colaci, Verónica Alfie, Sebastian Garcia Marti, Federico Augustovski, Andres Pichon Riviere, Andrea Alcaraz

## Abstract

**Introduction:**

Health technology assessments (HTAs) are essential for evidence-informed decision-making. In urgent clinical scenarios, ultra-rapid HTAs provide concise evaluations to guide patient-specific decisions in 72 hours. The Institute for Clinical Effectiveness and Health Policy (IECS), with over 20 years of experience in HTA, has played a key role in delivering these rapid assessments to support efficient healthcare decisions in Argentina.

**Methods:**

A descriptive analysis was conducted using the registry of ultra-rapid HTA reports produced by the IECS from April 2012 to August 2024. Two independent researchers extracted data from the historical database where the data on requests are stored. The database contains information about the requesters, type of technology, pathology, request dates, and delivery dates. The researchers categorized the information by type of technology (drugs, devices, procedures, diagnostics), therapeutic area (surgery, cardiovascular, infections, neurology, endocrinology, metabolic disorders, etc.), disease type (rare or not), and the requester of the report. The data were analyzed quantitatively.

**Results:**

Over 12 years, IECS produced 544 ultra-rapid HTA reports. The most requested therapeutic areas were oncology (n=124; 23%), neurology (n=94; 17%), musculoskeletal (n=45; 8%), other (n=39; 7%), and ophthalmology (n=31; 6%). Evaluated technologies primarily included drugs (n=255; 47%), followed by procedures (n=152; 28%), devices (n=70; 13%), and diagnostic tests (n=67; 12%). Reports on orphan diseases represented 19 percent of the total. Most requests were submitted by social health insurance providers (n=413; 76%), private insurers (n=123; 23%), hospitals (n=7; 1%), and the Ministry of Health (n=1).

**Conclusions:**

The IECS analyzed 544 ultra-rapid HTA reports, showing a growing demand for timely evaluations, especially in oncology and neurology. Only 19 percent focused on orphan diseases, with most addressing prevalent conditions. These findings underscore the need for rapid assessment to support decision-making by social health insurers, improve access to critical technologies, and achieve a more equitable, transparent, and fair health system.

